# Alterations of gut microbiota are associated with brain structural changes in the spectrum of Alzheimer's disease: the SILCODE study in Hainan cohort

**DOI:** 10.3389/fnagi.2023.1216509

**Published:** 2023-07-14

**Authors:** Beiqi He, Can Sheng, Xianfeng Yu, Liang Zhang, Feng Chen, Ying Han

**Affiliations:** ^1^School of Biomedical Engineering, Hainan University, Haikou, China; ^2^Department of Neurology, The Affiliated Hospital of Jining Medical University, Jining, China; ^3^Department of Neurology, Xuanwu Hospital of Capital Medical University, Beijing, China; ^4^Department of Radiology, Hainan General Hospital (Hainan Affiliated Hospital of Hainan Medical University), Haikou, China; ^5^Center of Alzheimer's Disease, Beijing Institute for Brain Disorders, Beijing, China; ^6^National Clinical Research Center for Geriatric Disorders, Beijing, China

**Keywords:** Alzheimer's disease, gut microbiota, 16S ribosomal RNA, brain structural, magnetic resonance imaging

## Abstract

**Background:**

The correlation between gut microbiota and Alzheimer's disease (AD) is increasingly being recognized by clinicians. However, knowledge about the gut–brain–cognition interaction remains largely unknown.

**Methods:**

One hundred and twenty-seven participants, including 35 normal controls (NCs), 62 with subjective cognitive decline (SCD), and 30 with cognitive impairment (CI), were included in this study. The participants underwent neuropsychological assessments and fecal microbiota analysis through 16S ribosomal RNA (rRNA) Illumina Miseq sequencing technique. Structural MRI data were analyzed for cortical anatomical features, including thickness, sulcus depth, fractal dimension, and Toro's gyrification index using the SBM method. The association of altered gut microbiota among the three groups with structural MRI metrics and cognitive function was evaluated. Furthermore, co-expression network analysis was conducted to investigate the gut–brain–cognition interactions.

**Results:**

The abundance of *Lachnospiraceae, Lachnospiracea_incertae_sedis, Fusicatenibacter*, and *Anaerobutyricum* decreased with cognitive ability. *Rikenellaceae, Odoribacteraceae*, and *Alistipes* were specifically enriched in the CI group. *Mediterraneibacter* abundance was correlated with changes in brain gray matter and cerebrospinal fluid volume (*p* = 0.0214, *p* = 0.0162) and significantly with changes in cortical structures in brain regions, such as the internal olfactory area and the parahippocampal gyrus. The three colonies enriched in the CI group were positively correlated with cognitive function and significantly associated with changes in cortical structure related to cognitive function, such as the precuneus and syrinx gyrus.

**Conclusion:**

This study provided evidence that there was an inner relationship among the altered gut microbiota, brain atrophy, and cognitive decline. Targeting the gut microbiota may be a novel therapeutic strategy for early AD.

## 1. Introduction

Alzheimer's disease (AD) is the most common form of dementia, imposing a heavy economic and social burden worldwide. Currently, the pathogenesis of AD remains unclear. Due to the lack of effective treatments in the stage of AD dementia, exploring new mechanisms of AD may be vital for providing successful therapeutic strategies (Chowdhary et al., 2021; Gauthier et al., 2021).

Recently, gut microbiota may be a critical factor in developing AD. Gut microbiota can interact with individual brain physiological activity through the microbiota–gut–brain axis, leading to the development of a variety of neurodegenerative diseases, such as AD and Parkinson's disease (Main and Minter, 2017; Cryan et al., 2019). Current studies have demonstrated that the structure of intestinal microbiota in patients with AD and mild cognitive impairment (MCI) is altered compared with cognitively normal adults (Cattaneo et al., 2017; Zhuang et al., 2018; Liu et al., 2019). In addition, our previous studies have also confirmed the similar alterations of specific gut microbiota, such as phylum *Firmicutes*, phylum *Bacteroidetes*, and their corresponding genus, in subjective cognitive decline (SCD) and amyloid-β (Aβ) positive cognitively normal individuals (Sheng et al., 2021, 2022), providing the preliminary evidence of altered gut microbiota in preclinical AD. Furthermore, these altered gut microbiotas are found to be associated with cognitive function and global brain Aβ burden (Chen et al., 2020; Li et al., 2020). Kim et al. confirmed that transplantation of fecal microbiota of wild-type mice into transgenic mice model of AD could improve Aβ plaque formation and cognitive impairment. Thus, alterations of gut microbiota are involved in the pathogenesis of the AD mice model (Kim et al., 2020). However, knowledge about the gut–brain interaction remains largely unknown.

A growing body of evidence suggests several pathways potentially connecting the intestinal microbiota and brain, including neuroimmune, gut microbiota-derived metabolites, neurotransmitters, and enteroendocrine signaling. The intestinal microbiota may influence brain amyloidosis and central nervous system (CNS) homeostasis through these substances or influence neural messages carried by the vagal and spinal afferent neurons, further leading to the development of AD (Kowalski and Mulak, 2019; Mahmoudian Dehkordi et al., 2019; Fung, 2020). However, studies describing the effect of gut microbiota on brain structural and functional changes in AD are few.

Recent advances in brain imaging have provided an opportunity for elucidating the gut–brain–cognition interactions. The evidence of brain volume atrophy and cortex lesions in AD is commonly shown using structural magnetic resonance imaging (sMRI) (Vemuri and Jack, 2010; Whitwell, 2018). De Santis et al. (2019) proposed a possible framework called radiomicrobiomics, which highlighted that the combination of gut microbiota with brain imaging techniques will greatly enhance our understanding of the microbiota–gut–brain axis in regulating cognition in AD (Cryan et al., 2019). Based on the amygdala-based functional connectivity and voxel-based morphometry (VBM) analysis, Zheng et al. (2020) revealed that the disrupted distribution of genus *Roseburia* regulated the amygdala-based functional connectivity in patients with end-stage renal disease. One recent study reported that gut bacteria *Odoribacter* was positively associated with hippocampal volume, which might be mediated by acetic acids (Liang et al., 2022). However, current studies focusing on the interactions between gut microbiota, brain, and cognition in AD, especially in the whole spectrum of AD are still limited.

This study aimed to (1) explore the characteristics of intestinal microbiota in the spectrum of AD in the Hainan cohort; (2) investigate the correlation between gut microbiota and brain atrophy; and (3) elucidate the interrelationship between gut microbiota, brain structure, and cognitive function using co-expression network analysis.

## 2. Materials and methods

### 2.1. Participants

One hundred and twenty-seven participants were recruited between November 2021 and July 2022 for the Sino Longitudinal Study on Cognitive Decline (SILCODE) (Li et al., 2019). Participants in the present study were recruited from Memory Clinic in both Hainan General Hospital and Hainan Cancer Hospital, including normal control (NC) (*n* = 35), individuals with SCD (*n* = 62), and patients with cognitive impairment (CI) (MCI, *n* = 16; mild AD dementia, *n* = 14). The criteria for individuals with NC were as follows: (1) objective neuropsychological assessments within the normal range, adjusted for age, gender, and years of education; and (2) without complaints of cognitive decline or concerns about cognitive decline. Individuals with SCD were diagnosed according to the criteria proposed by Jessen et al. (2014, 2020), which were as follows: (1) self-experienced, persistent cognitive decline, mainly in the memory domain but not in other cognitive domains, which was not related to the acute event; (2) the onset was within 5 years; (3) issues associated with SCD; (4) the objective neuropsychological examination was within the normal range, adjusted for age, gender, and years of education; (5) failure to meet the diagnostic criteria for MCI or AD dementia (Jessen et al., 2014, 2020). The definition of MCI was in accordance with the criteria proposed by Jak and Bondi in 2014. Participants were considered to have MCI if any one of the following three criteria were met: (1) impaired scores (defined as >1 SD below the age/education-corrected normative means) on both measures in at least one cognitive domain (memory, language, or speed/executive function); (2) impaired scores in each of the three cognitive domains (memory, language, or speed/executive function); (3) Functional Activity Questionnaire (FAQ) ≥ 9 (Bondi et al., 2014). The diagnosis of AD dementia was based on the frameworks of the Diagnostic and Statistical Manual of Mental Disorders (fifth edition) and the National Institute on Aging—Alzheimer's Association (NIA-AA) workgroups (McKhann et al., 2011; Association American Psychiatric, 2013). In our study, patients with MCI and mild AD were defined as individuals with CI.

The exclusion criteria were as follows: (1) those with left-handedness or double-handedness; (2) those suffered from cerebrovascular or psychiatric disorders or other neurological disorders that may lead to cognitive impairment or congenital intellectual disability; (3) those with an experience of traumatic brain injury that may lead to cognitive impairment; (4) those with severe sensory impairment or infectious disease that cannot complete the examination; (5) those who cannot undergo MRI scan, such as having metal implants or claustrophobia; (6) those with a history of taking probiotics, prebiotics, synbiotics, antibiotics, or medications to regulate gut microbiota within the last 3 months; (7) those with a history of long-term use of corticosteroids, immunosuppressants, or immunostimulatory drugs; and (8) those suffered from severe gastrointestinal disorders, such as irritable bowel syndrome, inflammatory bowel disease, and severe digestive and absorption abnormalities.

All clinical information was collected according to standard procedures as experienced neurologists and memory clinic specialists prescribed. Research activities in this study were conducted following the ethical standards of the Declaration of Helsinki. The Medical Research Ethics Committee and Institutional Review Board of Xuanwu Hospital at Capital Medical University approved them. Written informed consent was obtained at the time when participants were recruited.

### 2.2. Neuropsychological assessments

Demographic information was collected on age, gender, and years of education. An experienced neurologist interviewed each participant and their informant about their essential physical condition, recorded the assessment of their self-perceived abilities, and sought confirmation from their informants.

All participants carried on the following neuropsychological tests: (1) memory domain: Auditory Verbal Learning Test—Huashan version (AVLT-H) (Zhao et al., 2012); (2) executive domain: Shape Trails Test (STT-A and B) (Zhao et al., 2013); (3) language domain: Animal Fluency Test (AFT) (Guo et al., 2007) and Boston Naming Test (BNT) (Guo, 2006); (4) global cognitive function: Montreal Cognitive Assessment—Basic (MoCA-B) (Chen et al., 2016), Mini-Mental State Examination (MMSE) (Li et al., 2016), Memory and Executive Screening Scale (MES) (Guo et al., 2012), and Everyday Cognition (Ecog) (Farias et al., 2008); (5) daily functional activities: FAQ (González et al., 2022); (6) emotional state: Hamilton Depression Rating Scale (HAMD), Hamilton Anxiety Rating Scale (HAMA), and Geriatric Depression Scale (GDS) (Aikman and Oehlert, 2001); (7) sleep state: Pittsburgh Sleep Quality Index (PSQI) (Buysse et al., 1989), REM sleep behavior disorder screening questionnaire (RBDSQ) (Nomura et al., 2015), and Epworth Sleepiness Scale (ESS) (Johns, 1991); (8) Clinical symptom: Clinical Dementia Rating (CDR) (Lim et al., 2005).

### 2.3. Fecal sample collection and DNA extraction

In this study, fecal sample collection and preservation operations were carried out by the standardized fecal procedure proposed by the International Human Microbiome Standard (IHMS) and the Human Microbiome Project (HMP) (https://www.hmpdacc.org).

According to the instructions, the QIAamp DNA Stool Mini Kit (Qiagen, Hilden, Germany) was used to extract DNA from the fecal samples in a Class II biosafety laboratory. After extraction, the DNA concentration was quantified using the UV microspectrophotometer Thermo Nano-Drop 2000 (Thermo Scientific, MA, USA). The total DNA was checked for quality by 1% agarose gel electrophoresis. Finally, after assessing the DNA integrity and fragment size, the qualified DNA extracts were suspended in H_2_O and stored at −80°C prior to subsequent analysis.

### 2.4. 16s rRNA gene amplicon and sequencing

The 16S rRNA amplification region selected for this study is the V3-V4, using universal primers (341F and 806R) linked with indices and sequencing adaptors. The forward primer (5′-3′) was CCTAC GGGRSGCAGCAG (341F), and the reverse primer (5′-3′) was GGACTACVVGGGTATCTAATC (806R). The 5′ end of the universal primers was added to fit the Illumina NovaSeq PE250 sequencing of the splice and index sequences. The diluted genomic DNA was used as a template for PCR amplification using the KAPA HiFi Hotstart Ready Mix PCR kit high-fidelity enzyme. The 2% agarose gel electrophoresis was used to examine PCR products, and then the PCR product recovery was completed using the AxyPrep DNA Gel Extraction kit (AXYGEN). Amplified samples were subjected to library quality control using a Thermo Nano-Drop 2000 UV microspectrophotometer and 2% agarose gel electrophoresis. Qualified samples were subjected to library quantification using Qubit and homogenized according to the data available for each sample.

The completed amplified DNA samples were sequenced using the Illumina NovaSeq PE250 platform, resulting in paired-end sequencing (PE250) data. Paired-end reads from the double-end sequencing were spliced into a single sequence using PANDAseq software-long reads with a high variation region. The final clean reads were selected to be in the range of 250–500 nt.

### 2.5. Sequence analysis

In this study, the clean reads that completed the quality check were subjected to chimera removal operations and cluster analysis using the USEARCH tool. For clustering analysis, the clustering results were judged using a 97% similarity threshold to obtain operational taxonomic units (OTUs), each considered as a species representative (Edgar, 2010). For clean reads with identical sequences, the singletons were filtered out and matched to the OTU sequences one by one, and those that can be matched to the OTUs were output as mapped reads. All sequences were randomly sampled and leveled based on sufficient sequencing depth to avoid bias in analysis results due to differences in the quality of sequencing data among samples. The sequence with the highest abundance value in each OTU was selected as its representative sequence and compared to the sequences of known species in the 16S database for similarity, completing the species annotation of OUT. The 16S database used in this study is the RDP database (http://rdp.cme.msu.edu/index.jsp) (Cole et al., 2014).

The alpha and beta diversity in this study was assessed using QIIME software. The alpha diversity indices were used to assess the alpha diversity, and five alpha diversity indices (Chao1, Observed species, Simpson, Shannon, and PD_whole_tree) were selected for analysis in our study. Beta diversity was assessed by calculating the phylogenetic distance of the UniFrac values, which were divided into Weighted UniFrac concerning sequence abundance and Unweighted UniFrac without reference to sequence abundance.

This study uses the Kruskal.test function in R studio's stats tool package to analyze the inter-group differences in microbiota structure. First, the linear discriminant analysis (LDA) typical method was used by LEfSe, a cloud tool of the National Microbial Science Data Center (https://nmdc.cn/analyze/detailsid=600676b70b38496ee0c90921), as the processing platform, to estimate the influence of each species' abundance on the differences among groups and analyze the microbiota information and enrichment that had a significant response to the sample grouping information. Then, the significant difference information of the distribution of bacteria between different groups was screened using the rank sum test method, and the microbiota with the significant difference in relative abundance value between groups was obtained. The threshold value was *p* < 0.05 and was corrected by a false discovery rate (FDR).

### 2.6. Structural MRI scan and data preprocessing

The MRI data acquisition equipment involved in this study was a 3-T MR imager (Magnetom Trio Tim; Siemens, Erlangen, Germany) at the Radiology Department of Hainan General Hospital, using a magnetization-prepared rapid acquisition gradient-echo sequence to acquire three-dimensional T1-weighted images at the sagittal plane. T1-weighted imaging (T1W1) acquisition parameters: matrix = 256 × 256, FOV = 256 × 256 mm^2^, gap = 0, layer thickness = 1 mm, number of layers = 192, flip angle = 12°, TE = 2.9 ms, TR = 6.7 ms, TI = 450 ms, and voxel = (1 mm)3. The participants were asked to close their eyes and enter a quiet state without falling asleep and to keep their torsos as motionless as possible until the scan was completed.

The raw image data required a format conversion using Dcm2nii software to convert the DICOM format to the Nifti format required by the SPM 12 software. It was followed by preprocessing on MATLAB (R 2012b, MathWorks, Natick, MA, USA) using SPM 12 and the CAT 12 toolkit, including origin and bias field inhomogeneity correction. It segments the images into different gray matter, white matter, and cerebrospinal fluid volume (CSF) data files by CAT 12′s AMAP method. The images were normalized by the DARTEL algorithm function in the toolkit, aligned sample by sample image to a template of 555 healthy subjects in the IXI database (http://www.brain-development.org), and smoothed (Rajapakse et al., 1997). Total surface area (TSA), total intracranial volume (TIV), and actual and relative volumes of gray matter, white matter, and CSF (relative volumes were calculated by dividing each component volume by TIV) were calculated using the VBM method (Ashburner and Friston, 2000). The analysis of cortical morphology required re-aligning the samples with the DK40 template using algorithmic functions from the CAT 12 toolkit and the SBM method to complete the analysis of cortical anatomical features, including thickness, sulcus depth, fractal dimension, and Toro's gyrification index (Riccelli et al., 2017).

### 2.7. Statistical analysis

The statistical analysis was performed by IBM SPSS software (version 23, IBM, Armonk, NY, USA). For all intra-group statistical and inter-group comparison work on grouped data, the Shapiro–Wilk test (test of normality) and Levene's test (chi-square test) were performed before selecting the appropriate parametric or non-parametric test, respectively. A one-way ANOVA test was used for analyses of inter-group variance for continuous, normally distributed data, and the Kruskal–Wallis test for continuous, non-normally distributed data. The Pearson chi-square test was used to analyze inter-group variability for categorical variables.

When calculating correlations among the altered gut microbiota abundance, the structural index, and the neuropsychological assessments, biased correlation analysis was used through a linear mixed-effects model with age, years of education, and TIV as covariates, the aim of which was to exclude the influence of other confounding factors on the results. GraphPad Prism (version 9.0, GraphPad, San Diego, CA, USA) was used to describe the correlation.

Using Cytoscape to perform Spearman correlation analysis on the abundance of the screened gut microbiota, cortical index, volumetric index, and cognitive-related neuropsychological assessments and reflect significant interrelationships (*p* < 0.05), the results were tested by FDR to estimate the relationship between the pathways represented by the above feature values as a Network diagram for overall representation.

## 3. Results

### 3.1. Demographic and neuropsychological assessments

The results of demographic information and neuropsychological assessments of the NC, SCD, and CI participants are shown in Table 1. For all three groups of participants, there were no statistical differences in age, gender, or years of education (*p* > 0.05). There were also no statistical differences among the three groups in PSQI, RBDSQ, and ESS (*p* > 0.05). In the results of the three scales assessing mood (HAMD, HAMA, GDS), the differences among the three groups were statistically significant (*p* = 0.026, *p* = 0.017, and *p* = 0.049, respectively), and the SCD group had the highest scores in both HAMD and HAMA. There were extreme statistically significant differences (*p* < 0.001) in MMSE, MoCA-B, and FAQ scales, and the CI group had the most severe cognitive impairment on each of the scales.

**Table 1:** Demographic and neuropsychological assessments for all participants.

**Characteristics**	**Total (*n* = 127)**	**Group**			***p*-value**
		**NC (*****n*** = **35)**	**SCD (*****n*** = **62)**	**CI (*****n*** = **30)**	
Sex (F/M)	83/44	24/11	41/21	18/12	0.757
Age (y)	69 (65, 72)	69 (65, 71.5)	67 (65, 71)	71.5 (66, 77)	0.102
Education (y)	14 (12, 16)	14 (12, 16)	15 (12, 16)	12 (10.25, 13.75)	0.139
HAMD	5.28 ± 4.46	3.57 ± 3.53	6.06 ± 4.80	5.63 ± 4.31	0.026
HAMA	6.41 ± 5.37	4.23 ± 4.46	7.29 ± 5.06	7.13 ± 6.34	0.017
GDS	3.20 ± 2.61	2.31 ± 1.92	3.40 ± 2.87	3.80 ± 2.57	0.049
PSQI	5.19 ± 3.58	5.00 ± 3.64	5.69 ± 3.93	4.37 ± 2.58	0.236
RBDSQ	1.74 ± 1.81	1.69 ± 1.81	1.92 ± 1.88	1.43 ± 1.70	0.477
ESS	6.02 ± 4.73	4.51 ± 3.74	6.29 ± 4.29	7.23 ± 6.14	0.056
MMSE	26.72 ± 4.38	28.77 ± 1.48	28.03 ± 2.14	21.60 ± 5.96	< 0.001
AVLT-H N5	5.71 ± 3.46	8.31 ± 2.04	6.00 ± 3.00	2.07 ± 2.46	< 0.001
AVLT-H N7	20.46 ± 3.92	22.83 ± 1.52	21.08 ± 2.72	16.43 ± 4.91	< 0.001
STT-A	83.87 ± 41.70	70.57 ± 25.02	68.55 ± 30.66	131.07 ± 42.73	< 0.001
STT-B	172.91 ± 65.26	137.40 ± 35.34	152.60 ± 39.77	256.33 ± 64.09	< 0.001
AFT	19.24 ± 6.79	22.54 ± 5.08	20.44 ± 5.15	12.93 ± 7.52	< 0.001
BNT	25.48 ± 4.84	26.77 ± 2.75	26.85 ± 2.79	21.13 ± 7.13	< 0.001
MES	85.72 ± 15.92	92.49 ± 5.83	91.23 ± 6.99	66.47 ± 21.35	< 0.001
FAQ	1.87 ± 4.96	0.06 ± 0.24	0.74 ± 1.48	0.74 ± 1.48	< 0.001
Ecog	1.55 ± 0.63	1.18 ± 0.24	1.48 ± 0.44	2.12 ± 0.85	< 0.001
MoCA-B	24.13 ± 5.66	26.86 ± 2.38	25.84 ± 2.41	17.43 ± 7.68	< 0.001
CDR	0.22 ± 0.56	0 ± 0	0 ± 0	0.82 ± 0.15	< 0.001

NC, normal control; SCD, subjective cognitive decline; CI, cognitive impairment; M, male; F, female; y, year; HAMD, Hamilton Depression Rating Scale; HAMA, Hamilton Anxiety Rating Scale; GDS, Geriatric Depression Scale; PSQI, Pittsburgh Sleep Quality Index; RBDSQ, REM Sleep Behavior Disorder Questionnaire; ESS, Epworth Sleeping Scale; MMSE, Mini-Mental State Examination; AVLT-H N5, Auditory Verbal Learning Test—long-delayed recall; AVLT-H N7, Auditory Verbal Learning Test—recognition; STT-A, Shape Trails Test Part A; STT-B, Shape Trails Test Part B; AFT, Animal Fluency Test; BNT, Boston Naming Test; MES, Memory and Executive Screening Scale; FAQ, Functional Activities Questionnaire; Ecog, Everyday Cognition; MoCA-B, Montreal Cognitive Assessment—Basic; CDR, Clinical Dementia Rating.

### 3.2. The overall gut microbial communities among the three groups

**Figure 1:**
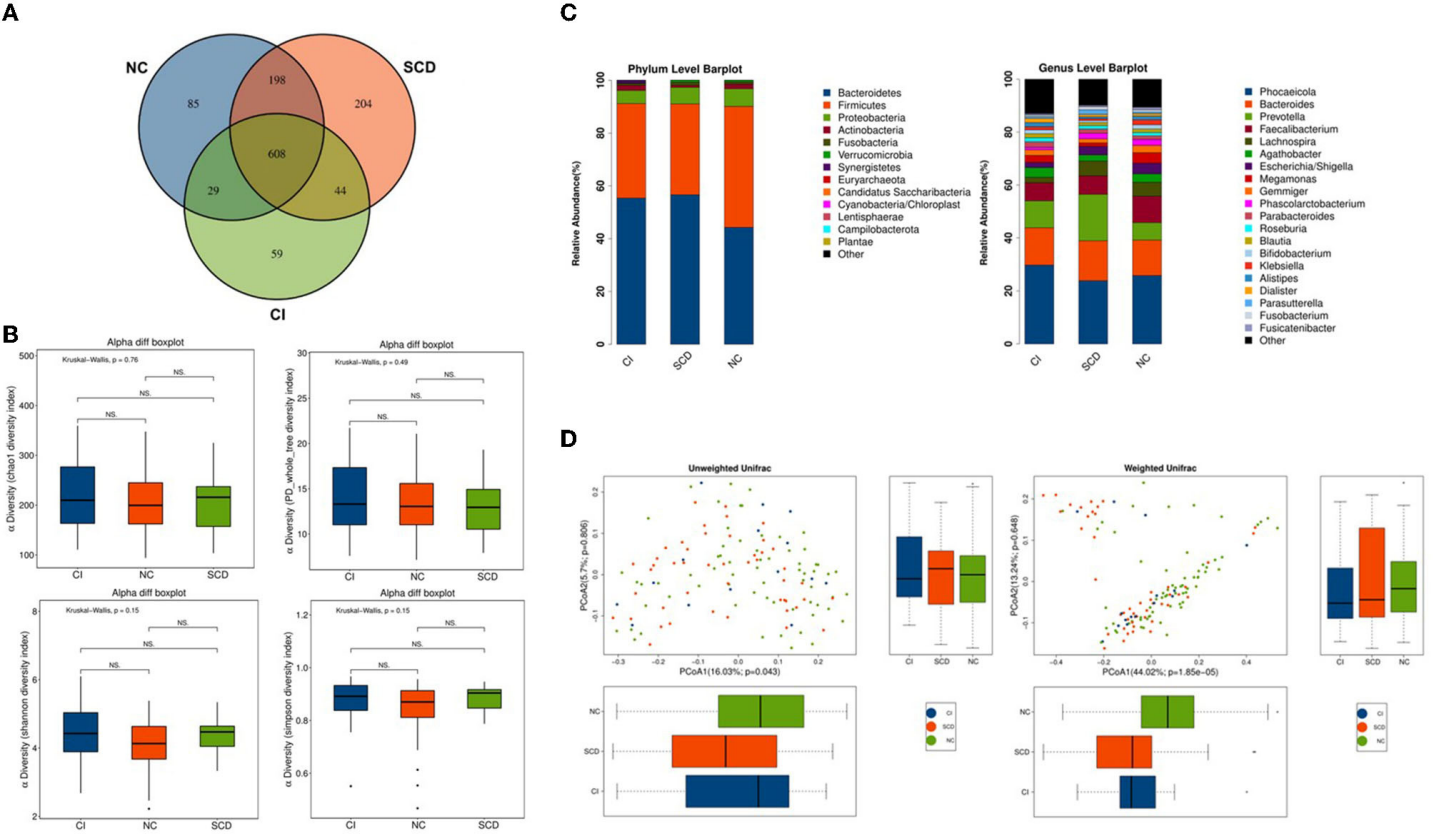
The overall structure of the gut microbiota based on the analysis of microbial diversity among NC, SCD, and CI. **(A)** Venn diagram illustrated the overlap of the OTUs identified in the gut microbiota among the three groups. **(B)** The alpha diversity of gut microbiota among the three groups. Each box plot represented the median, interquartile range, minimum, and maximum values. *P*-values were determined using the Kruskal–Wallis test. There were no significant differences in Chao1, PD_whole_tree, Shannon, and Simpson indices among the three groups. **(C)** The beta diversity among unweighted Unifrac and weighted Unifrac of the three groups using the principal coordinates analysis (PCoA). **(D)** Bar graphs indicate the bacterial community among the three groups at phylum and genus taxonomic levels.

A total of 1,227 OTUs were analyzed from the fecal samples collected from all the participants, of which 198 OTUs were shared between the NC and SCD groups, 44 OTUs were shared between the SCD and CI groups, and 608 OTUs were shared between the three groups (Figure 1A). A total of 13 phyla, 26 orders, 44 families, 74 families, and 216 genera of microorganisms were detected in all OTUs. The accumulation curves for all the detected OTU crop species showed a sharp rise followed by a gentle rise, indicating that the sampling operation parameters were set correctly, and the sampling was adequate in the pre-treatment (Supplementary Figure 1).

In the Kruskal–Wallis test for the six alpha diversity indices among the three groups, chao 1, PD_whole_tree, Shannon, and Simpson showed no statistical difference in the between-group analysis (*p* = 0.76, *p* = 0.49, *p* = 0.15, *p* = 0.15; *p* > 0.05) (Figure 1B). In this study, the PCoA method was used to cluster the species composition of the samples for both weighted and unweighted UniFrac values, and the results showed no significant clustering between samples for species diversity among the groups (Figure 1C), and no significant clustering between samples using the NMDS method (Supplementary Figure 2).

In this study, all samples detected were analyzed for species annotation and abundance, and the distribution of the dominant phylum and the composition of the dominant genus were consistent with the results of previous studies on the ecological structure of human-derived intestinal microbiota, as observed in the overall sample microbiota ecological structure (Supplementary Figure 3). The study analyzed the three groups of samples at different biological taxonomic levels (Figure 1D; Supplementary Figure 4). The qualitative and non-quantitative histograms show that at the phylum level, the phylum *Firmicutes* was enriched in the NC group. In contrast, the abundance of the phylum *Firmicutes* was significantly lower, and the abundance of the phylum *Bacteroidetes* was significantly higher in the CI group compared with the NC group. At the genus level, the beneficial intestinal bacteria such as *Faecalibacterium* and *Lachnospiracea* were enriched in the NC group compared with the CI group. The abundance of these groups was reduced in comparison with the CI group.

### 3.3. Alterations of the gut microbiota among the three groups

**Figure 2:**
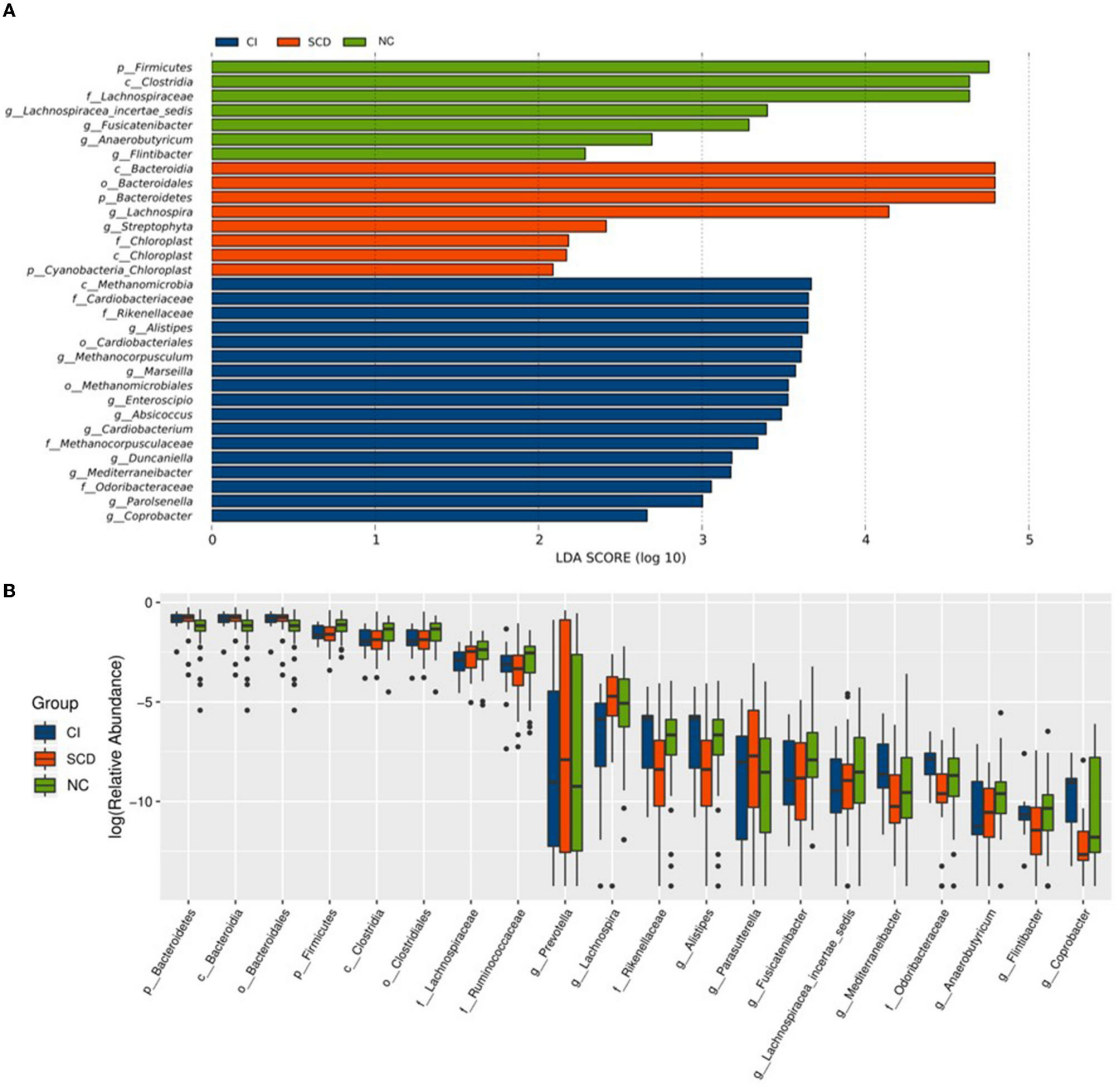
Alterations of the gut microbiota among the three groups. **(A)** The LEfSe analysis among NC, SCD, and CI. As shown in the histogram of LDA scores for differentially abundant taxa, the LDA scores indicated the enrichment of taxa in the NC group (green), the LDA scores indicated the enriched taxa in the SCD group (red), and the LDA scores indicated the enriched taxa in the CI group (blue). The LDA scores (log10) > 2 and *p* < 0.05 were listed. **(B)** The difference analysis of rank sum test among NC, SCD, and CI. The abscissa is the differential taxa, and the ordinate is the relative abundance log2, with different colors representing different groups.

In this study, to accurately know the information about the groups that were significantly different among the three groups, LEfSe analysis was used to assess the magnitude of the effect of the abundance of different species on the effect of group differences, to identify the groups or species that had a significant differential effect on the group information. As shown in Figure 2A, a total of thirty-two species were screened for the presence of statistical differences between groups at different biological taxonomic levels, of which seven were specifically enriched in the NC group, eight were specifically enriched in the SCD group, and seventeen were specifically enriched in the CI group. To further identify the groups that differed in abundance between groups, the rank sum test was used to analyze the significance of differences between groups, as shown in Figure 2B. Twenty groups (including all biological taxonomic classes) differed significantly among the groups, the phylum *Firmicutes*, class *Clostridia*, order *Clostridiales*, family *Lachnospiraceae*, genus *Fusicatenibacter*, genus *Lachnospiracea_incertae_sedis*, and *Anaerobutyricum* showed a progressively decreased prevalence from NC to SCD and CI; compared with both CI and SCD subjects, the NC population demonstrated a significant decrease in the abundance of phylum *Bacteroidetes*, class *Bacteroidia*, and order *Bacteroidales*.

Based on the results of the analysis of the differences between the three groups and the biological background of the screened groups, it was decided to retain nine distinct groups for the study. Among them, *Lachnospiraceae* (LDA = 4.64), *Lachnospiracea_incertae_sedis* (LDA = 3.40), *Fusicatenibacter* (LDA = 3.29), and *Anaerobutyricum* (LDA = 2.69) had decreasing abundance with cognitive ability and a decreasing trend between groups; the abundance of family *Rikenellaceae* (LDA = 3.65) and *Odoribacteraceae* (LDA = 3.06), and genus *Mediterraneibacter* (LDA = 3.18) and *Alistipes* (LDA = 3.65) in individuals with SCD were significantly reduced compared with others and enriched in the CI group.

### 3.4. Correlation between altered gut microbiota and brain structural features

In this study, sMRI image data from 100 participants acquired using a 3.0 T MRI machine were analyzed by the VBM method to obtain eight volume metrics, including the actual volume of each group of TIV, TSA, gray matter, white matter, and CSF (GM_abs, WM_abs, CSF_abs) and the relative volume of comparison individual TIV (GM_rel, WM_ rel, CSF_rel). As shown in Table 2, CSF_abs indicated statistical inter-group difference (*p* = 0.013); TSA displayed a significantly inter-group difference (*p* = 0.004); WM_abs, GM_abs, CSF_rel, WM_rel, and GM_rel were shown extremely statistically inter-group difference (*p* < 0.001); there was no statistically significant difference among the groups in TIV (*p* = 0.093). There are consistent with previous relevant studies.

**Table 2:** Statistical table of sMRI volume indices calculated by VBM.

**The indices of volumetry**	**Total (*n* = 100)**	**Groups**			***p*-value**
		**NC (*****n*** = **25)**	**SCD (*****n*** = **51)**	**CI (*****n*** = **24)**	
CSF_abs	341.9750 ± 70.0590	343.3282 ± 66.5699	325.3959 ± 62.5599	375.7958 ± 78.6731	0.013
WM_abs	497.3981 ± 60.2879	512.9503 ± 67.7562	508.8576 ± 52.2553	456.8464 ± 51.4591	< 0.001
GM_abs	613.3541 ± 57.34508	627.9692 ± 61.2396	626.0984 ± 47.0597	571.0485 ± 54.4706	< 0.001
CSF_rel	0.2349 ± 0.0398	0.2308 ± 0.0324	0.2219 ± 0.0328	0.2667 ± 0.0442	< 0.001
WM_rel	0.3422 ± 0.0244	0.3452 ± 0.0227	0.3485 ± 0.0210	0.3259 ± 0.0263	< 0.001
GM_rel	0.4228 ± 0.0240	0.4240 ± 0.0209	0.4295 ± 0.0212	0.4074 ± 0.0263	< 0.001
TSA	1,838.7473 ± 156.9746	1,871.1383 ± 175.8909	1,865.9549 ± 131.4432	1,747.1906 ± 157.1386	0.004
TIV	1,452.7272 ± 134.3649	1,484.2477 ± 159.9418	1,460.3519 ± 121.3357	1,403.6906 ± 123.6676	0.093

NC, normal control; SCD, subjective cognitive decline; CI, cognitive impairment; CSF_abs, cerebrospinal fluid's absolute value; WM_abs, white matter's absolute value; GM_abs, gray matter's absolute value; CSF_rel, cerebrospinal fluid's relative tissue volume; WM_rel, white matter's relative tissue volume; GM_rel, gray matter's relative tissue volume; TSA, total surface area; TIV, total intracranial volume.

Participants' cortical morphology was analyzed using SBM, during which four participants' data that could not be aligned with the DK40 template were censored. Four cortical indices (thickness, sulcus depth, fractal dimension, and Toro's gyrification index) were calculated for the 72 brain regions delineated. Due to the plethora of results, Supplementary Table 1 presents the cortical index statistics for the 43 specific brain regions correlated with the characteristic microbiotas in subsequent analysis. As shown in the table, these regions reveal statistical inter-group differences among the three groups: the entorhinal, the lateral prefrontal, the medial orbitofrontal, the temporal pole, the pars orbitalis, the superior parietal, and the supramarginal gyrus, particularly in the thickness of entorhinal, lateral orbitofrontal, temporal pole, the sulcus depth of pars orbitalis, the Toro's gyrification index of superior parietal, and supramarginal.

**Figure 3:**
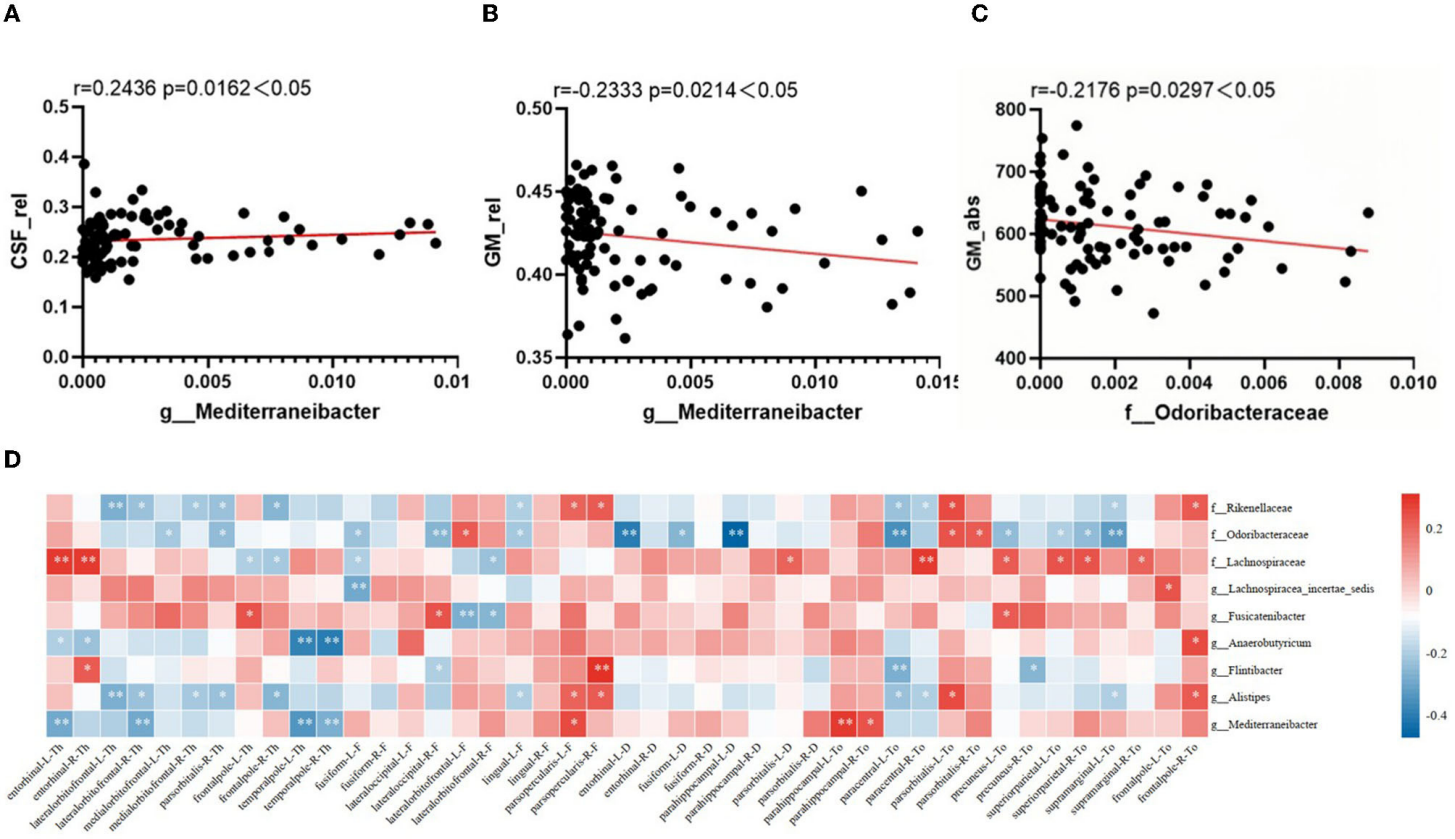
Correlation between altered characteristic taxa and brain structural features. **(A–C)** The result of correlation analysis between characteristic taxa abundance and sMRI volume index. **(A)** Scatter chart of correlation analysis between the abundance of *Mediterraneibacter* and cerebrospinal fluid's relative tissue volume. **(B)** Scatter chart of correlation analysis between the abundance of *Mediterraneibacter* and gray matter's relative tissue volume. **(C)** Scatter chart of correlation analysis between the abundance of *Odoribacteraceae* and gray matter's absolute value. **(D)** Heatmap of correlation between characteristic GM abundance and sMRI cortical index. The correlation coefficients (Corr) are displayed. Red or blue signifies a positive or negative correlation, respectively. *p < 0.05, **p < 0.01.

Correlations between sMRI brain volume metrics and characteristic microbiota abundance were performed using partial correlation analysis, adjusted for age, years of education, and TIV. As shown in Figures 3A–C, the *Mediterraneibacter*'s abundance was positively correlated with CSF_rel (*r* = 0.2436, *p* = 0.0162) and negatively correlated with GM_rel (*r* = −0.2333, *p* = 0.0214); the *Mediterraneibacter*'s abundance was negatively correlated with GM_abs (*r* = −0.2176, *p* = 0.0297); and the *Odoribacteraceae*'s abundance was negatively correlated with GM_abs (*r* = −0.2176, *p* = 0.0297).

The correlation between characteristic microbiota and brain cortex structure is shown in Figure 3D, with characteristic microbiota abundance correlating to varying degrees with several cortical indicators in multiple brain regions. As visualized in the heatmap, the *Rikenellaceae*'s abundance was negatively correlated with thickness of the lateral orbitofrontal cortex in both right and left brain (*r* = −0.286, *p* = 0.005; *r* = −0.239, *p* = 0.021); the *Odoribacteraceae*'s abundance was negatively correlated with sulcus depth of the left parahippocampal gyrus (*r* = −0.475, *p* < 0.001), with Toro's gyrification index of the left precuneus (*r* = −0.256, *p* = 0.013), with Toro's gyrification index of the superior parietal cortex in both right and left brain (*r* = −0.207, *p* = 0.046; *r* = −0.246, *p* = 0.018), and with Toro's gyrification index of the left supramarginal cortex (*r* = −0.32, *p* = 0.002); the *Lachnospiraceae*'s abundance was positively correlated with thickness of the entorhinal cortex in both right and left brain (*r* = 0.277, *p* = 0.007; *r* = 0.282, *p* = 0.006), with Toro's gyrification index of the left precuneus (*r* = 0.217, *p* = 0.037), and with Toro's gyrification index of the superior parietal cortex in both right and left brain (*r* = 0.242, *p* = 0.02; *r* = 0.231, *p* = 0.026); the *Lachnospiracea_ incertae_sedis*'s abundance was negatively correlated with fractal dimension of the left fusiform gyrus (*r* = −0.296, *p* = 0.004); the *Fusicatenibacter*'s abundance was negatively correlated with fractal dimension of the left lateral orbitofrontal cortex (*r* = −0.286, *p* = 0.006); the *Anaerobutyricum*'s abundance was negatively correlated with thickness of the temporal pole in both right and left brain (*r* = −0.389, *p* < 0.001; *r* = −0.41, *p* < 0.001); the *Alistipes*'s abundance was negatively correlated with thickness of the left lateral orbitofrontal cortex (*r* = −0.286, *p* = 0.005); and the Mediterraneibacter's abundance was negatively correlated with thickness of the left entorhinal cortex (*r* = −0.292, *p* = 0.004), with the thickness of temporal pole in both right and left brain (*r* = −0.335, *p* = 0.001; *r* = −0.28, *p* = 0.007), and positively correlated with Toro's gyrification index in the left parahippocampal gyrus (*r* = 0.28, *p* = 0.006).

### 3.5. Network analysis among altered gut microbiota, neuroimaging features, and cognition

**Figure 4:**
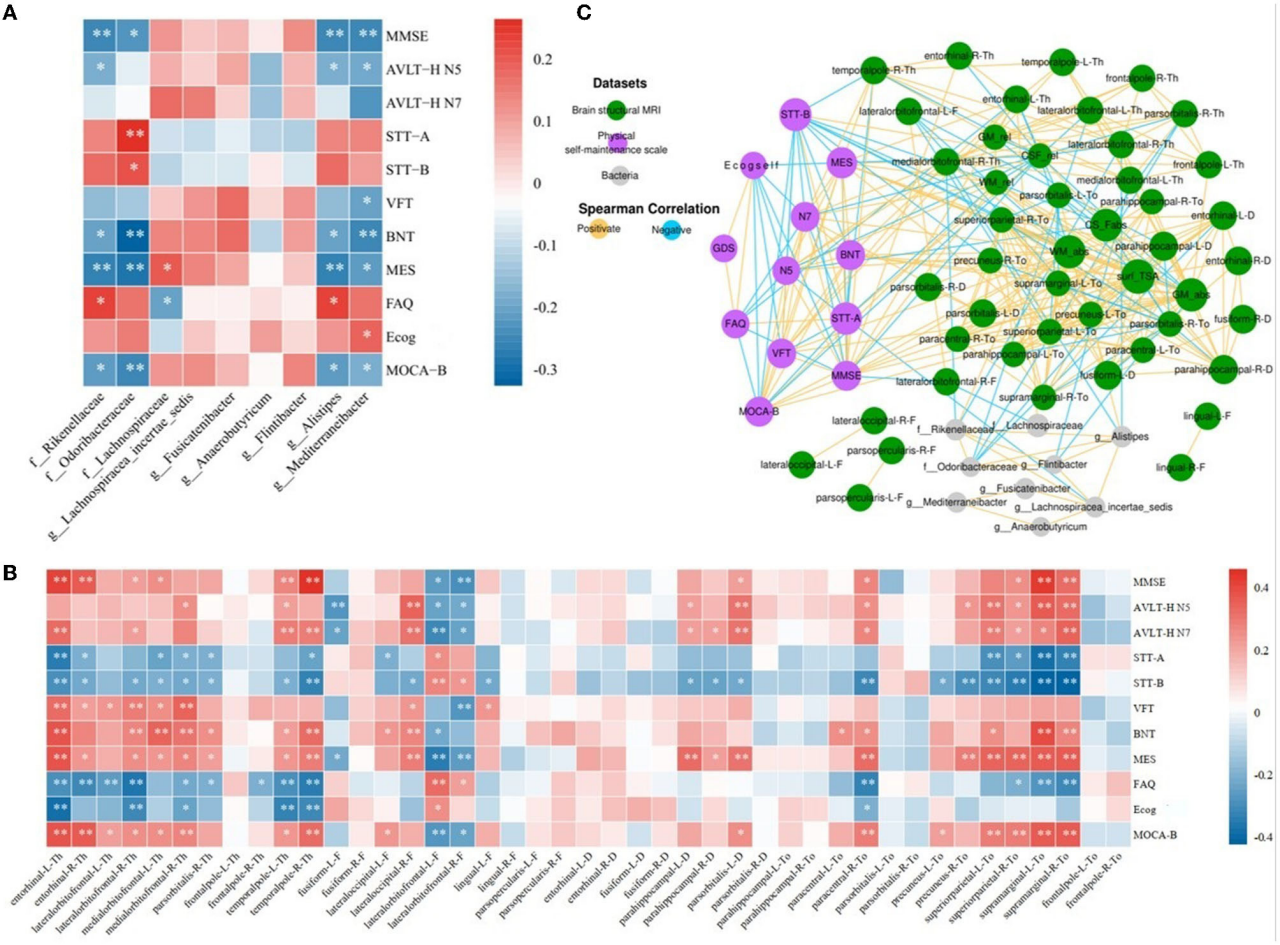
Network analysis among characteristic taxa, neuroimaging features, and cognition. **(A)** Heatmap of correlations between the night characteristic taxa and neuropsychological assessments. **(B)** Heatmap of correlation between the neuropsychological assessments and sMRI cortical index. **(C)** Significant associations among characteristic taxa, neuroimaging features, and neuropsychological assessments. The Spearman correlation was used to calculate pairwise correlations of all the measurements. The network shows significant correlations (FDR < 0.05) between each pair of measurement types. The size of nodes represents the number of connections with others. Orange edge, Spearman correlation coefficient > 0; blue edge, Spearman correlation coefficient < 0. *p < 0.05, **p < 0.01.

Using a linear mixed-effects model, we analyzed the correlations between the nine abundance values of distinct groups and the results of the eleven neuropsychological scales after excluding the effects of age and education (Figure 4A). The abundance of *Mediterraneibacter* and *Alistipes* all correlated with the results of the different tables. The abundance of *Rikenellaceae* indicated a positive correlation with the FAQ results (*r* = 0.228, *p* = 0.011) and negative correlations with the results of MMSE, MES, AVLT-H N5, BNT, and MoCA-B (*r* < 0, *p* < 0.05). The abundance of *Odoribacteraceae* indicated positive correlations with STT-A and STT-B results (*r* > 0, *p* < 0.05) and negative correlations with BNT, MES, MoCA-B, and MMSE results (*r* < 0, *p* < 0.05). The abundance of *Lachnospiraceae* indicated a positive correlation with MES (*r* = 0.189, *p* = 0.036) and a negative correlation with the FAQ results (*r* = −0.206, *p* = 0.022). The abundance of *Alistipes* indicated a positive correlation with the FAQ results (*r* = 0.228, *p* = 0.011) and negative correlations with the MMSE, MES, AVLT-H N5, BNT, and MoCA-B results (*r* < 0, *p* < 0.05). The abundance of *Mediterraneibacter* performed a positive correlation with Ecog (*r* = 0.183, *p* = 0.041) and negative correlations with MMSE, MES, AVLT-H N5, BNT, VFT, and MoCA-B results (*r* < 0, *p* < 0.05).

After implementing a linear mixed-effects model and regressing the effects of age and years of education, the results of the correlation analysis between 43 cortical indicators and the results of 11 neuropsychological scales were obtained. As shown in Figure 4B, cortical thickness in the entorhinal, the lateral orbitofrontal, the medial orbitofrontal, and the temporal pole, the cortical fractal dimension of the lateral occipital, the sulcus depth of the parahippocampal gyrus and pars orbitalis, and the cortical Toro's gyrification index of the paracentral, precuneus, superior parietal, and supramarginal gyrus showed positive correlations with MMSE, AVLT-H, VFT, BNT, MES, and MoCA-B results (*r* > 0, *p* < 0.05) and negative correlations with STT, FAQ, and Ecog results (*r* < 0, *p* < 0.05). The fractal dimension of the fusiform and lateral orbitofrontal showed positive correlations with STT, FAQ, and Ecog results (*r* > 0, *p* < 0.05) and negative correlations with MMSE, AVLT-H, VFT, BNT, MES, and MoCA-B results (*r* < 0, *p* < 0.05).

The results of the screened nine characteristic microbiota abundances, 43 cortical indicators, seven volumetric indicators, and neuropsychological assessment scales were subjected to an interaction network correlation analysis using Cytoscape to understand the interaction of colony characteristics and further explain the mechanism of formation of differences between characteristic colony groups. There was a tight correlation between the three feature values, forming a complete network of mechanisms (Figure 4C).

## 4. Discussion

In this study, we found nine specific gut microbiota that possibly related to the development of AD, of which four were negatively correlated with cognitive function, and one was positively correlated with cognitive function. In addition, three specificities were enriched in the cognitively impaired population. The joint analysis of the abundance of nine specific microbiota and individual brain structure sMRI image biomarkers found a significant correlation. *Mediterraneibacter* abundance was positively correlated to CSF and negatively correlated to gray matter volume. Moreover, we found a significant correlation between specific gut microbiota abundance and changes in cortical structure in brain regions related to cognitive function, such as entorhinal, fusiform, parahippocampal, precuneus, and superior parietal.

The gut microbiota compositions in the whole spectrum of AD were similar to that in previous studies, with the phylum *Firmicutes* enriched in the NC group and the phylum *Bacteroides* enriched in the CI group at the phylum level. In the study of Vogt et al. (2017) the abundance of phylum *Firmicutes* in AD was significantly lower than that of healthy controls, and the abundance of phylum *Bacteroides* was significantly increased. Similarly, the decreased *Firmicutes* in the AD population was also confirmed in a cross-sectional study with the research cohort of the Chinese population (Liu et al., 2019). Decreased Firmicutes promote the production of toxic metabolites and pro-inflammatory cytokines, resulting in fewer beneficial substances for intestinal stability, damaging the intestinal epithelial barrier, and subsequently causing the blood–brain barrier dysfunction through peripheral circulation and neuroinflammation, which finally cause brain lesions (Bhat and Kapila, 2017; Cerovic et al., 2019; Liu et al., 2020). There were no significant differences in intra-group alpha diversity analysis and between-group beta diversity analysis and noticeable clustering effect among the three groups. It may be due to a decrease in the number of beneficial bacteria in the intestine and an increase in pathogenic bacteria as the disease progresses. In brief, compared with previous studies based on the Xuanwu cohort (Sheng et al., 2021, 2022), there were common and specific gut microbiota changes in the Hainan cohort. In addition to individual differences in the gut flora, we considered that the regional differences may also influence the distribution of individual gut microbiota.

From the perspective of whole brain volume, SCD shows gray matter and white matter atrophy similar to that in AD in brain regions, such as the hippocampus, medial temporal lobe, anterior cuneiform, and temporoparietal region (Sun et al., 2016, 2019; Hu et al., 2019). In addition, *Mediterraneibacter* was found to be correlated with regional brain structural indices. Some studies have reported that *Mediterraneibacter* and *Odoribacteraceae* participate in bile acid spectrum metabolism and indirectly regulate many individual functions, such as intestinal barrier, neuroinflammation, and immune response (Kim et al., 2019; Sato et al., 2021). Three studies have demonstrated that bile acid can affect metabolism and immune response by regulating Th17 and promoting the production and regulation of homeostasis by Treg cells in the colon tissue (Campbell et al., 2020; Song et al., 2020; Paik et al., 2022). In AD studies, serum concentrations of primary bile acids were significantly reduced in AD patients compared with cognitively normal older adults, while levels of secondary bile acids and deoxycholic acids produced by gut microbiota were elevated. It suggests that potential gut microbiota dysregulation in AD patients may be caused by increased gut colonization by anaerobic bacteria capable of dehydroxylation of primary bile acids (Mahmoudian Dehkordi et al., 2019).

At present, it has been proved that short-chain fatty acids (SCFA) can inhibit Aβ deposition by interfering with the assembly of Aβ40 and Aβ42 polypeptides into soluble and neurotoxic Aβ aggregates *in vitro* (Ho et al., 2018). In a study of elderly cohorts, the level of individual gut microbiota-derived SCFA was negatively correlated with the pathological degree of Aβ deposition in the brain (Marizzoni et al., 2020). Studies on AD have shown that butyric acid at the gut–blood barrier (GBB) interface promotes the antimicrobial activity of intestinal macrophages, limits bacterial translocation, and enhances intestinal barrier function by providing energy to intestinal epithelial cells and increasing connection integrity (Mathewson et al., 2016). At the blood–brain barrier (BBB) interface, SCFA, mainly butyrate, increases the expression of tight junction proteins in the frontal cortex and hippocampus in mice and acts as a histone deacetylase inhibitor, transcriptional regulator, and anti-inflammatory molecule to maintain brain microvascular homeostasis (Braniste et al., 2014; Chriett et al., 2019). The characteristic microbiota *Lachnospiracea_incertae_ sedis, Fusicatenibacter*, and *Anaerobutyricum* in the phylum *Firmicutes* analyzed in our study were all SCFA-producing bacteria. In this report, *Lachnospiraceae* was positively correlated with cortical thickness in the entorhinal zone, the Toro's gyrification index in the precuneus zone, and the Toro's gyrification index in the superior parietal, and these indicators were also positively correlated with the degree of cognitive impairment. The abundance of Lachnospiraceae and *Lachnospiracea_ incertae_sedis* was negatively correlated with the fractal dimension of the left fusiform cortex, and *Fusicatenibacter* was negatively correlated with the fractal dimension of the lateral prefrontal cortex. Moreover, the three cortical indicators of the two brain regions also showed an inverse correlation with the neuropsychological assessments. In the study of Thaker et al. (2017) the severity of Aβ deposition in the brain of AD patients and the severity of pathological changes in NFT are positively correlated with the thickness of the entorhinal cortex. The meta-analysis study of the pars orbitalis cortex by Belyk et al. (2017) found that as a semantic information-processing brain region, it is also involved in processing emotional perception signals and belongs to the convergence area of the functional and dynamic perception network. The precuneus is part of the posterior parietal cortex inside the cerebral hemisphere, and its cognitive function involves episodic memory, subspace, self-related information processing, metacognition, and consciousness processes. In a study for early AD therapy, researchers significantly improved episodic memory through stimulation of the precuneus (Koch et al., 2018).

Several microbiotas associated with gastrointestinal and psychiatric disorders have been reported to colonize the gut specifically in AD patients, and this condition affects cognitive function in individuals with AD (Mitrea et al., 2022; Rønnow Sand et al., 2022). *Rikenellaceae, Odoribacteraceae*, and *Alistipes* were specifically enriched in the intestines of individuals with CI in our study. The abundance was significantly correlated with the degree of cognitive impairment. In their correlation studies with individual cortical indicators, the primary brain areas affected were the frontal pole, fusiform, lingua, parahippocampal, precuneus, superior parietal, and supramarginal gyrus. However, there are few studies involving the impact of the abovementioned bacteria on brain structural alterations and cognitive impairment.

Currently, there are few studies demonstrating the interaction among gut microbiota, structural brain lesions, and individual cognitive function. In this study, we conducted a joint analysis of three types of characteristic indicators (characteristic GM abundance, sMRI imaging indices, and neuropsychological scale results), and the results showed that there were complex connections between various indicators, which further confirmed the existence of gut microbiota changes in early AD risk groups. In summary, it is suggested that these three microbiotas are related to the decline of individual cognitive function. Their increased abundance may affect the stable structure of specific brain regions, thereby impairing cognitive function. However, further experiments are needed to study the mediator substances and pathways of this effect.

There are several limitations to this study: (1) It is a single-center cross-sectional study of an elderly population in Hainan with a relatively small sample size; however, a larger sample size from multiple centers would be useful and necessary to provide more evidence. The results of this study will require a longitudinal study and randomized controlled trial of participants to determine whether the effect of the specific microbiota on disease progression is confounded by other factors. (2) The 16S rRNA high-throughput assay technique used in the current study achieves low resolution, is sensitive to the specific primers selected and the number of PCR cycles, and may be followed up with a macro-genome sequencing approach that extends taxonomic resolution to the species or strain level, as well as analyses potential functional information. (3) We have only confirmed that alterations in gut microbiota show effects on cognitive performance and brain structure, but the causal nature of the interaction between gut microbiota and cognitive function remains unclear. Subsequent inclusion of biomarkers such as gut microbiota metabolites and intra-blood cytokines will provide more comprehensive insights into the potential mechanisms that form a closed-loop study of gut–brain interactions in AD progression.

## 5. Conclusion

The present study characterized the gut microbiota and brain structure in the spectrum of AD. It confirmed the possible interactions of gut microbiota alterations on brain structure and cognitive function, providing a new perspective on the pathogenesis of AD and a potential target for AD treatments.

## Data availability statement

The datasets presented in this study can be found in online repositories. The names of the repository/repositories and accession number(s) can be found below: https://www.ncbi.nlm.nih.gov/bioproject/PRJNA984461.

## Ethics statement

The studies involving human participants were reviewed and approved by the Medical Ethics Committee of Xuanwu Hospital, Capital Medical University, Beijing, China. The patients/participants provided their written informed consent to participate in this study. Written informed consent was obtained from the individual(s) for the publication of any potentially identifiable images or data included in this article.

## Author contributions

BH and CS contributed to the conception and design of the study. BH organized the database, performed the statistical analysis, and wrote the first draft of the manuscript. CS wrote sections of the manuscript and contributed to manuscript revision. XY and LZ contributed to the manuscript revision. YH and FC contributed to the manuscript revision, read, and approved the submitted version. All authors contributed to the article and approved the submitted version.

## References

[B1] Aikman G. G. Oehlert M. E. (2001). Geriatric depression scale. J. Am. Geriatr. Soc. 22, 63–70. 10.1300/J018v22n03_07

[B2] Ashburner J. Friston K. J. (2000). Voxel-based morphometry–the methods. Neuroimage 11 (6 Pt 1), 805–821. 10.1006/nimg.2000.0582 10860804

[B3] Association American Psychiatric (2013). Diagnostic and Statistical Manual of Mental Disorders Fifth Edition (DSM-5.) Arlington, VA: American Psychiatric Pub.

[B4] Belyk M. Brown S. Lim J. Kotz S. A. (2017). Convergence of semantics and emotional expression within the IFG pars orbitalis. Neuroimage 156, 240–248. 10.1016/j.neuroimage.2017.04.020 28400265

[B5] Bhat M. I. Kapila R. (2017). Dietary metabolites derived from gut microbiota: critical modulators of epigenetic changes in mammals. Nutr. Rev. 75, 374–389. 10.1093/nutrit/nux001 28444216

[B6] Bondi M. W. Edmonds E. C. Jak A. J. Clark L. R. Delano-Wood L. McDonald C. R.. (2014). Neuropsychological criteria for mild cognitive impairment improves diagnostic precision, biomarker associations, and progression rates. J. Alzheimers Dis. 42, 275–289. 10.3233/JAD-140276 24844687 PMC4133291

[B7] Braniste V. Al-Asmakh M. Kowal C. Anuar F. Abbaspour A. Tóth M.. (2014). The gut microbiota influences blood-brain barrier permeability in mice. Sci. Transl. Med. 6, 263ra158. 10.1126/scitranslmed.3009759 25411471 PMC4396848

[B8] Buysse D. J. Reynolds C. F. 3rd Monk T. H. Berman S. R. Kupfer D. J. (1989). The Pittsburgh Sleep Quality Index: a new instrument for psychiatric practice and research. Psychiatry Res. 28, 193–213. 10.1016/0165-1781(89)90047-4 2748771

[B9] Campbell C. McKenney P. T. Konstantinovsky D. Isaeva O. I. Schizas M. Verter J.. (2020). Bacterial metabolism of bile acids promotes generation of peripheral regulatory T cells. Nature 581, 475–479. 10.1038/s41586-020-2193-0 32461639 PMC7540721

[B10] Cattaneo A. Cattane N. Galluzzi S. Provasi S. Lopizzo N. Festari C.. (2017). Association of brain amyloidosis with pro-inflammatory gut bacterial taxa and peripheral inflammation markers in cognitively impaired elderly. Neurobiol. Aging 49, 60–68. 10.1016/j.neurobiolaging.2016.08.019 27776263

[B11] Cerovic M. Forloni G. Balducci C. (2019). Neuroinflammation and the gut microbiota: possible alternative therapeutic targets to counteract Alzheimer's disease? Front. Aging Neurosci. 11, 284. 10.3389/fnagi.2019.00284 31680937 PMC6813195

[B12] Chen C. Ahn E. H. Kang S. S. Liu X. Alam A. Ye K. (2020). Gut dysbiosis contributes to amyloid pathology, associated with C/EBPβ/AEP signaling activation in Alzheimer's disease mouse model. Sci. Adv. 6, eaba0466. 10.1126/sciadv.aba0466 32832679 PMC7439296

[B13] Chen K. L. Xu Y. Chu A. Q. Ding D. Liang X. N. Nasreddine Z. S.. (2016). Validation of the Chinese version of montreal cognitive assessment basic for screening mild cognitive impairment. J. Am. Geriatr. Soc. 64, e285–e290. 10.1111/jgs.14530 27996103

[B14] Chowdhary N. Barbui C. Anstey K. J. Kivipelto M. Barbera M. Peters R.. (2021). Reducing the risk of cognitive decline and dementia: WHO recommendations. Front. Neurol. 12, 765584. 10.3389/fneur.2021.765584 35082745 PMC8784726

[B15] Chriett S. Dabek A. Wojtala M. Vidal H. Balcerczyk A. Pirola L. (2019). Prominent action of butyrate over β-hydroxybutyrate as histone deacetylase inhibitor, transcriptional modulator and anti-inflammatory molecule. Sci. Rep. 9, 742. 10.1038/s41598-018-36941-9 30679586 PMC6346118

[B16] Cole J. R. Wang Q. Fish J. A. Chai B. McGarrell D. M. Sun Y.. (2014). Ribosomal Database Project: data and tools for high throughput rRNA analysis. Nucleic Acids Res. 42 (Database issue), D633–D642. 10.1093/nar/gkt1244 24288368 PMC3965039

[B17] Cryan J. F. O'Riordan K. J. Cowan C. S. M Sandhu K. V. Bastiaanssen T. F.. (2019). The microbiota-gut-brain axis. Physiol. Rev. 99, 1877–2013. 10.1152/physrev.00018.2018 31460832

[B18] De Santis S. Moratal D. Canals S. (2019). Radiomicrobiomics: advancing along the gut-brain axis through big data analysis. Neuroscience 403, 145–149. 10.1016/j.neuroscience.2017.11.055 29237568

[B19] Edgar R. C. (2010). Search and clustering orders of magnitude faster than BLAST. Bioinformatics 26, 2460–2461. 10.1093/bioinformatics/btq461 20709691

[B20] Farias S. T. Mungas D. Reed B. R. Cahn-Weiner D. Jagust W. Baynes K.. (2008). The measurement of everyday cognition (ECog): scale development and psychometric properties. J. Neuropsychol. 22, 531–544. 10.1037/0894-4105.22.4.531 18590364 PMC2877034

[B21] Fung T. C. (2020). The microbiota-immune axis as a central mediator of gut-brain communication. Neurobiol. Dis. 136, 104714. 10.1016/j.nbd.2019.104714 31846737

[B22] Gauthier S. RosaNeto P. Morais J. A. Webster C. (2021). World Alzheimer Report 2021: Journey Through the Diagnosis of Dementia. London: Alzheimer's Disease International.

[B23] González D. A. Gonzales M. M. Resch Z. J. Sullivan A. C. Soble J. R. (2022). Comprehensive evaluation of the functional activities questionnaire (FAQ) and its reliability and validity. J Assess. 29, 748–763. 10.1177/1073191121991215 33543638 PMC8339133

[B24] Guo Q. H. (2006). Boston naming test in Chinese Elderly, patient with mild cognitive impairment and Alzheimer's dementia. J. Chin. Mental Health J. 20, 81–84.

[B25] Guo Q. H. Jin L. L. Hong Z. (2007). A specific phenomenon of animal fluency test in Chinese elderly. J. Chinese Mental Health J. 21, 622–625. 10.1016/j.conbuildmat.2005.08.001

[B26] Guo Q. H. Zhou B. Zhao Q. H. Wang B. Hong Z. (2012). Memory and Executive Screening (MES): a brief cognitive test for detecting mild cognitive impairment. J. BMC Neurol. 12, 119. 10.1186/1471-2377-12-119 23050770 PMC3492138

[B27] Ho L. Ono K. Tsuji M. Mazzola P. Singh R. Pasinetti G. M. (2018). Protective roles of intestinal microbiota derived short chain fatty acids in Alzheimer's disease-type beta-amyloid neuropathological mechanisms. Exp. Rev. Neurother. 18, 83–90. 10.1080/14737175.2018.1400909 29095058 PMC5958896

[B28] Hu X. Teunissen C. E. Spottke A. Heneka M. T. Düzel E. Peters O.. (2019). Smaller medial temporal lobe volumes in individuals with subjective cognitive decline and biomarker evidence of Alzheimer's disease-Data from three memory clinic studies. Alzheimers Dement. 15, 185–193. 10.1016/j.jalz.2018.09.002 30321506

[B29] Jessen F. Amariglio R. E. Buckley R. F. van der Flier W. F. Han Y. Molinuevo J. L.. (2020). The characterisation of subjective cognitive decline. Lancet Neurol. 19, 271–278. 10.1016/S1474-4422(19)30368-0 31958406 PMC7062546

[B30] Jessen F. Amariglio R. E. van Boxtel M. Breteler M. Ceccaldi M. Chételat G.. (2014). A conceptual framework for research on subjective cognitive decline in preclinical Alzheimer's disease. Alzheimers Dement. 10, 844–852. 10.1016/j.jalz.2014.01.001 24798886 PMC4317324

[B31] Johns M. W. (1991). A new method for measuring daytime sleepiness: the Epworth sleepiness scale. Sleep. 14, 540–545. 10.1093/sleep/14.6.540 1798888

[B32] Kim J. S. Lee K. C. Suh M. K. Han K. I. Eom M. K. Lee J. H.. (2019). Mediterraneibacter butyricigenes sp. nov., a butyrate-producing bacterium isolated from human faeces. J. Microbiol. 57, 38–44. 10.1007/s12275-019-8550-8 30594982

[B33] Kim M. S. Kim Y. Choi H. Kim W. Park S. Lee D.. (2020). Transfer of a healthy microbiota reduces amyloid and tau pathology in an Alzheimer's disease animal model. Gut 69, 283–294. 10.1136/gutjnl-2018-317431 31471351

[B34] Koch G. Bonn,ì S. Pellicciari M. C. Casula E. P. Mancini M. Esposito R.. (2018). Transcranial magnetic stimulation of the precuneus enhances memory and neural activity in prodromal Alzheimer's disease. Neuroimage 169, 302–311. 10.1016/j.neuroimage.2017.12.048 29277405

[B35] Kowalski K. Mulak A. (2019). Brain-Gut-Microbiota Axis in Alzheimer's Disease. J. Neurogastroenterol. Motil. 25, 48–60. 10.5056/jnm18087 30646475 PMC6326209

[B36] Li H. Jia J. Yang Z. (2016). Mini-mental state examination in elderly Chinese: a population-based normative study. J. Alzheimers Dis. 53, 487–496. 10.3233/JAD-160119 27163822

[B37] Li X. Wang X. Su L. Hu X. Han Y. (2019). Sino Longitudinal Study on Cognitive Decline (SILCODE): protocol for a Chinese longitudinal observational study to develop risk prediction models of conversion to mild cognitive impairment in individuals with subjective cognitive decline. BMJ Open 9, e028188. 10.1136/bmjopen-2018-028188 31350244 PMC6661672

[B38] Li Z. Zhu H. Guo Y. Du X. Qin C. (2020). Gut microbiota regulate cognitive deficits and amyloid deposition in a model of Alzheimer's disease. J. Neurochem. 155, 448–461. 10.1111/jnc.15031 32319677

[B39] Liang X. Fu Y. Cao W. T. Wang Z. Zhang K. Jiang Z.. (2022). Gut microbiome, cognitive function and brain structure: a multi-omics integration analysis. Transl. Neurodegener. 11, 49. 10.1186/s40035-022-00323-z 36376937 PMC9661756

[B40] Lim W. S. Chin J. J. Lam C. K. Lim P. P. Sahadevan S. (2005). Clinical dementia rating: experience of a multi-racial Asian population. Alzheimers Dis. Assoc. Disord. 19, 135–142. 10.1097/01.wad.0000174991.60709.36 16118530

[B41] Liu P. Wu L. Peng G. Han Y. Tang R. Ge J.. (2019). Altered microbiomes distinguish Alzheimer's disease from amnestic mild cognitive impairment and health in a Chinese cohort. Brain Behav. Immun. 80, 633–643. 10.1016/j.bbi.2019.05.008 31063846

[B42] Liu S. Gao J. Zhu M. Liu K. Zhang H. L. (2020). Gut microbiota and dysbiosis in Alzheimer's disease: implications for pathogenesis and treatment. Mol. Neurobiol. 57, 5026–5043. 10.1007/s12035-020-02073-3 32829453 PMC7541367

[B43] Mahmoudian Dehkordi S. Arnold M. Nho K. Ahmad S. Jia W. Xie G.. (2019). Altered bile acid profile associates with cognitive impairment in Alzheimer's disease-An emerging role for gut microbiome. Alzheimers Dement. 15, 76–92. 10.1016/j.jalz.2018.07.217 30337151 PMC6487485

[B44] Main B. S. Minter M. R. (2017). Microbial immuno-communication in neurodegenerative diseases. Front. Neurosci. 11, 151. 10.3389/fnins.2017.00151 28386215 PMC5362619

[B45] Marizzoni M. Cattaneo A. Mirabelli P. Festari C. Lopizzo N. Nicolosi V.. (2020). Short-chain fatty acids and lipopolysaccharide as mediators between gut dysbiosis and amyloid pathology in Alzheimer's disease. J. Alzheimers Dis. 78, 683–697. 10.3233/JAD-200306 33074224

[B46] Mathewson N. D. Jenq R. Mathew A. V. Koenigsknecht M. Hanash A. Toubai T.. (2016). Gut microbiome-derived metabolites modulate intestinal epithelial cell damage and mitigate graft-versus-host disease. Nat. Immunol. 17, 505–513. 10.1038/ni.3400 26998764 PMC4836986

[B47] McKhann G. M. Knopman D. S. Chertkow H. Hyman B. T. Jack C. R. Jr Kawas, C. H.. (2011). The diagnosis of dementia due to Alzheimer's disease: recommendations from the National Institute on Aging-Alzheimer's Association workgroups on diagnostic guidelines for Alzheimer's disease. Alzheimers Dement. 7, 263–269. 10.1016/j.jalz.2011.03.005 21514250 PMC3312024

[B48] Mitrea L. Nemeş S. A. Szabo K. Teleky B. E. Vodnar D. C. (2022). Guts imbalance imbalances the brain: a review of gut microbiota association with neurological and psychiatric disorders. Front. Med. 9, 813204. 10.3389/fmed.2022.813204 35433746 PMC9009523

[B49] Nomura T. Inoue Y. Kagimura T. Kusumi M. Nakashima K. (2015). Validity of the Japanese version of the REM Sleep Behavior Disorder (RBD) Screening Questionnaire for detecting probable RBD in the general population. J. Psychiatry Clin. Neurosci. 69, 477–482. 10.1111/pcn.12286 25727855

[B50] Paik D. Yao L. Zhang Y. Bae S. D'Agostino G. D. Zhang M.. (2022). Human gut bacteria produce T(H)17-modulating bile acid metabolites. Nature 603, 907–912. 10.1038/s41586-022-04480-z 35296854 PMC9132548

[B51] Rajapakse J. C. Giedd J. N. Rapoport J. L. (1997). Statistical approach to segmentation of single-channel cerebral MR images. IEEE Trans. Med. Imaging 16, 176–186. 10.1109/42.563663 9101327

[B52] Riccelli R. Toschi N. Nigro S. Terracciano A. Passamonti L. (2017). Surface-based morphometry reveals the neuroanatomical basis of the five-factor model of personality. Soc. Cogn. Affect. Neurosci. 12, 671–684. 10.1093/scan/nsw175 28122961 PMC5390726

[B53] Rønnow Sand J. Troelsen F. S. Horváth-Puhó E. Henderson V. W. Sørensen H. T. Erichsen R. (2022). Risk of dementia in patients with inflammatory bowel disease: a Danish population-based study. Aliment. Pharmacol. Ther. 56, 831–843. 10.1111/apt.17119 35781292 PMC9545113

[B54] Sato Y. Atarashi K. Plichta D. R. Arai Y. Sasajima S. Kearney S. M.. (2021). Novel bile acid biosynthetic pathways are enriched in the microbiome of centenarians. Nature 599, 458–464. 10.1038/s41586-021-03832-5 34325466

[B55] Sheng C. Lin L. Lin H. Wang X. Han Y. Liu S. L. (2021). Altered gut microbiota in adults with subjective cognitive decline: the SILCODE Study. J. Alzheimers Dis. 82, 513–526. 10.3233/JAD-210259 34024839

[B56] Sheng C. Yang K. He B. Du W. Cai Y. Han Y. (2022). Combination of gut microbiota and plasma amyloid-β as a potential index for identifying preclinical Alzheimer's disease: a cross-sectional analysis from the SILCODE study. Alzheimers Res. Ther. 14, 35. 10.1186/s13195-022-00977-x 35164860 PMC8843023

[B57] Song X. Sun X. Oh S. F. Wu M. Zhang Y. Zheng W.. (2020). Microbial bile acid metabolites modulate gut RORγ(+) regulatory T cell homeostasis. Nature 577, 410–415. 10.1038/s41586-019-1865-0 31875848 PMC7274525

[B58] Sun Y. Dai Z. Li Y. Sheng C. Li H. Wang X.. (2016). Subjective cognitive decline: mapping functional and structural brain changes—A combined resting-state functional and structural MR imaging study. J. Radio. 281, 185–92. 10.1148/radiol.2016151771 27002419

[B59] Sun Y. Wang X. Wang Y. Dong H. Lu J. Scheininger T.. (2019). Anxiety correlates with cortical surface area in subjective cognitive decline: APOE ε4 carriers versus APOE ε4 non-carriers. Alzheimers Res. Ther. 11, 50. 10.1186/s13195-019-0505-0 31159873 PMC6547570

[B60] Thaker A. A. Weinberg B. D. Dillon W. P. Hess C. P. Cabral H. J. Fleischman D. A.. (2017). Entorhinal cortex: antemortem cortical thickness and postmortem neurofibrillary tangles and amyloid pathology. Am. J. Neuroradiol. 38, 961–965. 10.3174/ajnr.A5133 28279988 PMC5433913

[B61] Vemuri P. Jack C. R. Jr. (2010). Role of structural MRI in Alzheimer's disease. Alzheimers Res. Ther. 2, 23. 10.1186/alzrt47 20807454 PMC2949589

[B62] Vogt N. M. Kerby R. L. Dill-McFarland K. A. Harding S. J. Merluzzi A. P. Johnson S. C.. (2017). Gut microbiome alterations in Alzheimer's disease. Sci. Rep. 7, 13537. 10.1038/s41598-017-13601-y 29051531 PMC5648830

[B63] Whitwell J. L. (2018). Alzheimer's disease neuroimaging. Curr. Opin. Neurol. 31, 396–404. 10.1097/WCO.0000000000000570 29762152

[B64] Zhao Q. Guo Q. Li F. Zhou Y. Wang B. Hong Z. (2013). The Shape Trail Test: application of a new variant of the Trail making test. PLoS ONE 8, e57333. 10.1371/journal.pone.0057333 23437370 PMC3577727

[B65] Zhao Q. Lv Y. Zhou Y. Hong Z. Guo Q. (2012). Short-term delayed recall of auditory verbal learning test is equivalent to long-term delayed recall for identifying amnestic mild cognitive impairment. PLoS ONE 7, e51157. 10.1371/journal.pone.0051157 23236445 PMC3517417

[B66] Zheng L. J. Lin L. Zhong J. Zhang Z. Ye Y. B. Zhang X. Y.. (2020). Gut dysbiosis-influence on amygdala-based functional activity in patients with end stage renal disease: a preliminary study. Brain Imaging Behav. 14, 2731–2744. 10.1007/s11682-019-00223-3 32304020

[B67] Zhuang Z. Q. Shen L. L. Li W. W. Fu X. Zeng F. Gui L.. (2018). Gut microbiota is altered in patients with Alzheimer's Disease. J. Alzheimers Dis. 63, 1337–1346. 10.3233/JAD-180176 29758946

