# Supplementary material for: Alterations of gut microbiota are associated with brain structural changes in the spectrum of Alzheimer's disease: the SILCODE study in Hainan cohort
Source: Front Aging Neurosci. 2023 Jul 14;15:1216509. doi: 10.3389/fnagi.2023.1216509 (PMC10375500; doi:10.3389/fnagi.2023.1216509)
Supplement: Supplementary file 1 [file Data_Sheet_1.docx]

Supplementary Material

Article Title

Beiqi He^1^, Can Sheng^2,^ *, Xianfeng Yu^3^, Liang Zhang^1^, Feng Chen^6,^ *, Ying Han^1,3,4,5,^ *

*** Correspondence:**

Ying Han, MD, PhD, E-mail: hanying@xwh.ccmu.edu.cn; ORCID ID: 0000-0003-0377-7424

Can Sheng, MD, E-mail: canyeweiwu2013@163.com; ORCID ID: 0000-0002-5573-4082

Feng Chen, MD, PhD, E-mail: fenger0802@163.com; ORCID ID: 0000-0002-9129-7895

# Supplementary Figures and Tables


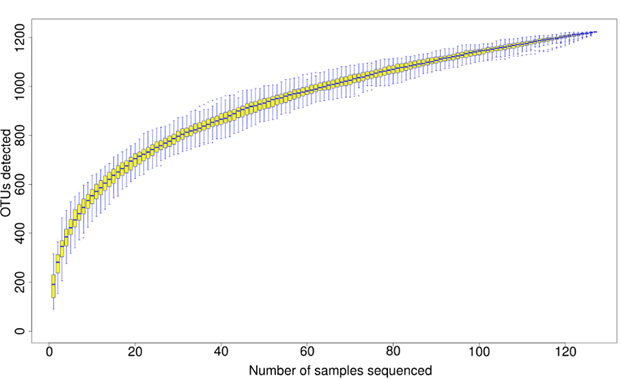
**Supplementary Figure 1.** Specaccum species accumulation curve.


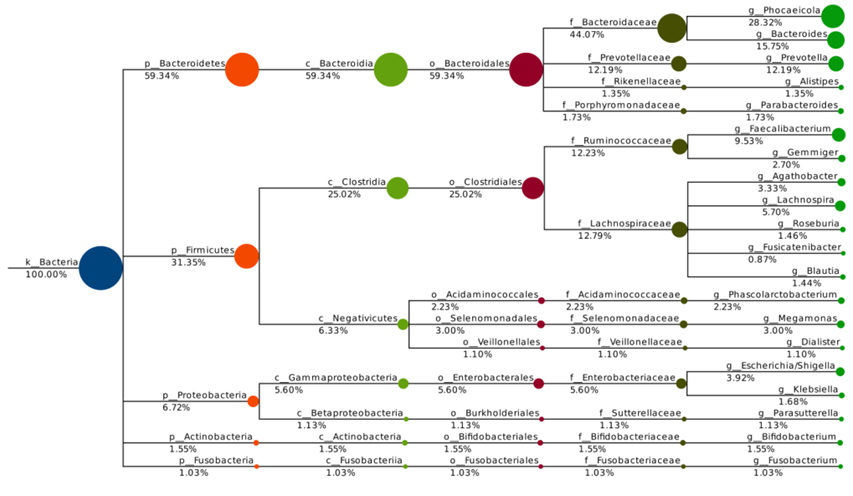
**Supplementary Figure 2.** Tree diagram of species classification and abundance ratio.


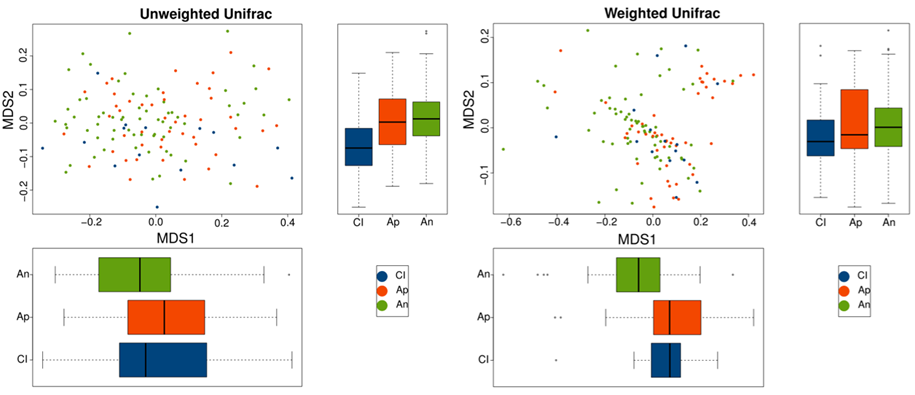
**Supplementary Figure 3.** NMDS analysis of beta diversity between groups.


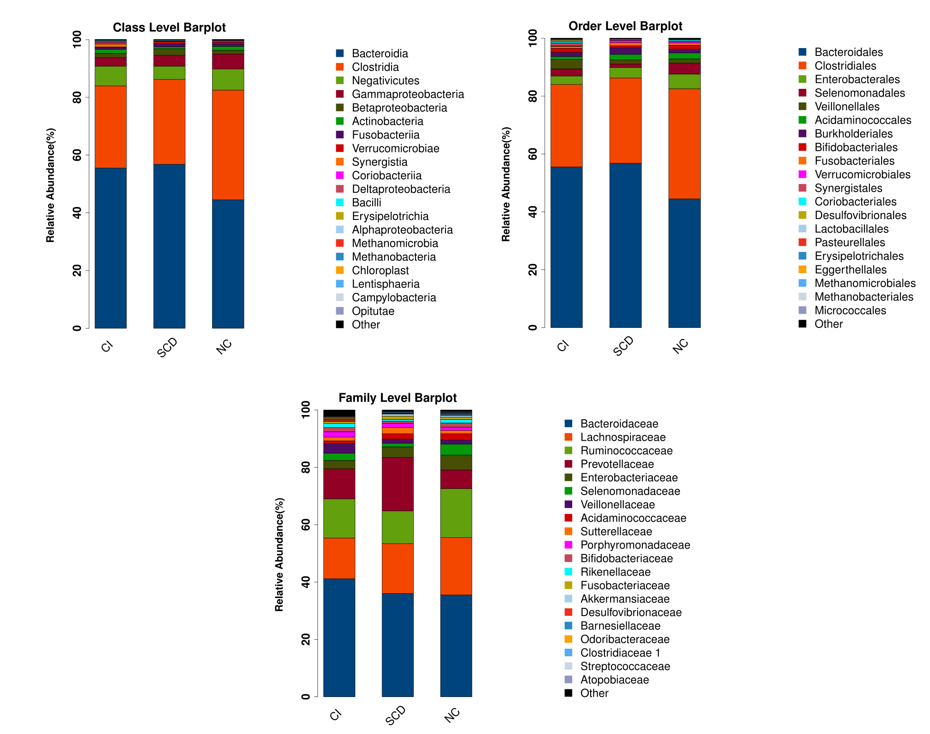
**Supplementary Figure 4.** The bacterial community among the three groups at class, order, and family taxonomic levels indicated by bar graphs.

**Supplementary Table 1.** Statistical table of sMRI cortical indexes in different brain regions calculated by SBM.

| The indices of cortical morphology | Total  (n=96) | Groups | | | p Value |
| --- | --- | --- | --- | --- | --- |
|  |  | NC  (n=24) | SCD  (n=48) | CI  (n=24) |  |
| entorhinal-L-Th | 3.3354 ± 0.3326 | 3.3766 ± 0.1892 | 3.4240 ± 0.3140 | 3.1171 ± 0.3897 | <0.001*** |
| entorhinal-R-Th | 3.4843 ± 0.3846 | 3.5107 ± 0.3759 | 3.5571 ± 0.3366 | 3.3121 ± 0.4421 | 0.034* |
| lateralorbitofrontal-L-Th | 2.5577 ± 0.1288 | 2.5931 ± 0.1168 | 2.5708 ± 0.1096 | 2.4962 ± 0.1572 | 0.019* |
| lateralorbitofrontal-R-Th | 2.5716 ± 0.1362 | 2.5957 ± 0.1011 | 2.5878 ± 0.1350 | 2.5152 ± 0.1569 | 0.061 |
| medialorbitofrontal-L-Th | 2.2392 ± 0.1312 | 2.2555 ± 0.1167 | 2.2680 ± 0.1126 | 2.1657 ± 0.1547 | 0.005** |
| medialorbitofrontal-R-Th | 2.3128 ± 0.1120 | 2.3404 ± 0.0957 | 2.3260 ± 0.1042 | 2.2589 ± 0.1275 | 0.020* |
| parsorbitalis-R-Th | 2.5099 ± 0.1485 | 2.5261 ± 0.1151 | 2.5313 ± 0.1464 | 2.4512 ± 0.1710 | 0.080 |
| frontalpole-L-Th | 2.4633 ± 0.1859 | 2.4627 ± 0.1605 | 2.4785 ± 0.1729 | 2.4334 ± 0.2334 | 0.629 |
| frontalpole-R-Th | 2.4045 ± 0.1770 | 2.4263 ± 0.1593 | 2.4112 ± 0.1688 | 2.3692 ± 0.2094 | 0.504 |
| temporalpole-L-Th | 3.3497 ± 0.3382 | 3.4287 ± 0.2594 | 3.3885 ± 0.3388 | 3.1932 ± 0.3679 | 0.027* |
| temporalpole-R-Th | 3.2907 ± 0.3851 | 3.3451 ± 0.2747 | 3.3969 ± 0.3815 | 3.0242 ± 0.3726 | <0.001*** |
| fusiform-L-F | 2.5067 ± 0.0883 | 2.4747 ± 0.0868 | 2.5204 ± 0.0824 | 2.5112 ± 0.0966 | 0.113 |
| fusiform-R-F | 2.5123 ± 0.0958 | 2.5151 ± 0.0793 | 2.5134 ± 0.1006 | 2.5073 ± 0.1044 | 0.956 |
| lateraloccipital-L-F | 2.4935 ± 0.0818 | 2.4789 ± 0.0726 | 2.5101 ± 0.0732 | 2.4749 ± 0.1011 | 0.135 |
| lateraloccipital-R-F | 2.4731 ± 0.0978 | 2.4849 ± 0.0952 | 2.4807 ± 0.0885 | 2.4462 ± 0.1157 | 0.294 |
| lateralorbitofrontal-L-F | 2.3905 ± 0.0620 | 2.3744 ± 0.0572 | 2.3879 ± 0.0611 | 2.4120 ± 0.0648 | 0.100 |
| lateralorbitofrontal-R-F | 2.3402 ± 0.0602 | 2.3372 ± 0.0414 | 2.3292 ± 0.0697 | 2.3652 ± 0.0490 | 0.053 |
| lingual-L-F | 2.6502 ± 0.0986 | 2.6546 ± 0.0969 | 2.6595 ± 0.0939 | 2.6273 ± 0.1097 | 0.416 |
| lingual-R-F | 2.6460 ± 0.0867 | 2.6471 ± 0.0802 | 2.6569 ± 0.0850 | 2.6230 ± 0.0950 | 0.297 |
| parsopercularis-L-F | 2.7910 ± 0.1474 | 2.7833 ± 0.1365 | 2.8080 ± 0.1640 | 2.7648 ± 0.1211 | 0.485 |
| parsopercularis-R-F | 2.9066 ± 0.1324 | 2.8887 ± 0.1180 | 2.9129 ± 0.1452 | 2.9119 ± 0.1223 | 0.749 |
| entorhinal-L-D | 7.8782 ± 1.2664 | 7.9534 ± 1.3756 | 8.0274 ± 1.1875 | 7.5047 ± 1.2855 | 0.244 |
| entorhinal-R-D | 7.2201 ± 1.4570 | 7.1991 ± 1.4122 | 7.4198 ± 1.5862 | 6.8417 ± 1.1832 | 0.286 |
| fusiform-L-D | 11.2897 ± 1.1297 | 11.2576 ± 1.1949 | 11.4061 ± 0.9701 | 11.0888 ± 1.3599 | 0.530 |
| fusiform-R-D | 10.0875 ± 1.0676 | 9.9805 ± 0.8396 | 10.2183 ± 1.1620 | 9.9331 ± 1.0820 | 0.486 |
| parahippocampal-L-D | 14.2100 ± 1.6130 | 14.5435 ± 1.8500 | 14.3805 ± 1.4430 | 13.5356 ± 1.5551 | 0.055 |
| parahippocampal-R-D | 14.0788 ± 1.5132 | 14.1269 ± 1.3490 | 14.3666 ± 1.5621 | 13.4551 ± 1.4390 | 0.052 |
| parsorbitalis-L-D | 5.2831 ± 0.7455 | 5.4483 ± 0.7692 | 5.3619 ± 0.6903 | 4.9602 ± 0.7614 | 0.043* |
| parsorbitalis-R-D | 5.3120 ± 0.7601 | 5.1999 ± 0.6452 | 5.4037 ± 0.8176 | 5.2407 ± 0.7536 | 0.494 |
| parahippocampal-L-To | 0.4192 ± 0.0194 | 0.4186 ± 0.0198 | 0.4228 ± 0.0178 | 0.4128 ± 0.0211 | 0.117 |
| parahippocampal-R-To | 0.4246 ± 0.0174 | 0.4257 ± 0.0187 | 0.4249 ± 0.0183 | 0.4228 ± 0.01456 | 0.831 |
| paracentral-L-To | 0.5263 ± 0.0227 | 0.5300 ± 0.0206 | 0.5265 ± 0.0222 | 0.5219 ± 0.0258 | 0.469 |
| paracentral-R-To | 0.5204 ± 0.0198 | 0.5234 ± 0.0185 | 0.5220 ± 0.0193 | 0.5142 ± 0.0213 | 0.197 |
| parsorbitalis-L-To | 0.5088 ± 0.0167 | 0.5083 ± 0.0166 | 0.5069 ± 0.0159 | 0.5132 ± 0.0183 | 0.327 |
| parsorbitalis-R-To | 0.5072 ± 0.0176 | 0.5122 ± 0.0226 | 0.5048 ± 0.0159 | 0.5068 ± 0.0146 | 0.245 |
| precuneus-L-To | 0.5002 ± 0.0143 | 0.5024 ± 0.0106 | 0.5013 ± 0.0137 | 0.4957 ± 0.0178 | 0.197 |
| precuneus-R-To | 0.4987 ± 0.0164 | 0.5007 ± 0.0181 | 0.4995 ± 0.0137 | 0.4950 ± 0.0194 | 0.421 |
| superiorparietal-L-To | 0.4870 ± 0.0187 | 0.4915 ± 0.0193 | 0.4890 ± 0.0161 | 0.4786 ± 0.0209 | 0.032* |
| superiorparietal-R-To | 0.4864 ± 0.0164 | 0.4884 ± 0.0173 | 0.4894 ± 0.0152 | 0.4785 ± 0.0158 | 0.022* |
| supramarginal-L-To | 0.5111 ± 0.0162 | 0.5142 ± 0.0163 | 0.5135 ± 0.0130 | 0.5032 ± 0.0195 | 0.020* |
| supramarginal-R-To | 0.5143 ± 0.0148 | 0.5184 ± 0.0130 | 0.5154 ± 0.0136 | 0.5081 ± 0.0171 | 0.040* |
| frontalpole-L-To | 0.4963 ± 0.0223 | 0.4951 ± 0.0206 | 0.4961 ± 0.0218 | 0.4981 ± 0.0256 | 0.895 |
| frontalpole-R-To | 0.4934 ± 0.0163 | 0.4997 ± 0.0151 | 0.4915 ± 0.0156 | 0.4908 ± 0.0178 | 0.087 |

**Table S1** *p<0.05, **p<0.01, ***p<0.001; cortical index names："-L" is "left brain", "-R" is "right brain"; "-Th" is the thickness of the cortex, "-F" is the fractal dimension of the cortex, "-D" is the sulcus depth of the cortex, and "-To" is the Toro's gyrification index of the cortex.
